# The genus *Scaptodrosophila* Duda (Diptera, Drosophilidae), part III: the *riverata* species group from China, with morphological and molecular evidence for five new species

**DOI:** 10.3897/zookeys.937.49794

**Published:** 2020-06-01

**Authors:** Yong Lin, Hong-Wei Chen

**Affiliations:** 1 Department of Entomology, South China Agricultural University, Tianhe, Guangzhou, 510642, China South China Agricultural University Guangzhou China

**Keywords:** cryptic species, DNA barcoding, integrative taxonomy, molecular research, new species group

## Abstract

A new species group, the *riverata* species group, is established within the genus *Scaptodrosophila* based on morphological and molecular evidence for five known and five new species from China: *S. abdentata***sp. nov.**, *S. cederholmi* (Okada, 1988), *S. crocata* (Bock, 1976), *S. paraclubata* (Sundaran & Gupta, 1991), *S. platyrhina***sp. nov.**, *S. puncticeps* (Okada, 1956), *S. riverata* (Singh & Gupta, 1977), *S. serrateifoliacea***sp. nov.**, *S. sinuata***sp. nov.** and *S. tanyrhina***sp. nov.** A key to this group is provided. Furthermore, 51 mtDNA *COI* sequences belonging to *S. puncticeps*, *S. riverata* and the five new species are used for verifying species boundaries defined by the morphological data.

## Introduction

A total of 12 species groups have been erected within the genus *Scaptodrosophila* (Duda 1923): *albifrontata* group (Wheeler and Takada 1966); *aterrima* group (Tsacas et al. 1988); *barkeri* group (Bock and Parsons 1978); *brunnea* group (Tsacas and Chassagnard 1976; Liu et al. 2017); *brunneipennis* group (Bock and Parsons 1978); *bryani* group (Throckmorton 1962); *coracina* group (Mather 1955; Liu and Chen 2018); *inornata* group (Parsons and Bock 1978); *latifasciaeformis* group (Burla 1954); *rufifrons* group (Papp et al. 1999); *saba* group (Burla 1954) and *victoria* group (Wheeler 1949). Together, these taxa include ca 300 species. Here we describe five new species from China that are morphologically similar to five known, yet unplaced, species: *S. cederholmi* (Okada, 1988) from Sri Lanka; *S. crocata* (Bock, 1976) from Australia; *S. paraclubata* (Sundaran & Gupta, 1991) from India; *S. puncticeps* (Okada, 1956) from China, Kuril Islands, Korea, Japan; and *S. riverata* (Singh & Gupta, 1977) from China, India, Myanmar. These ten species all have a yellowish-brown body; an arista with two dorsal and one ventral branch in addition to a terminal bifurcation; a large facial carina; and developed prescutellar setae. This morphological group is also supported by molecular data for the five new species and two of the five previously described taxa. The combined morphological and molecular evidence supports the establishment of a new species group, the *riverata* species group, based on five known and five new species from China.

DNA barcoding technology was employed to investigate the relationship of the *riverata* species group. Based on the results of the phylogenetic reconstruction, 51 barcode sequences of the *COI* (mitochondrial cytochrome *c* oxidase subunit I) gene belonging to two known and five new species were used to evaluate these species boundaries.

## Materials and methods

### Specimens

The *riverata* group species were collected by net sweeping from tussocks and tree trunks. All the examined specimens were preserved in 75% ethanol.

### Species identification

Total DNA was extracted from the abdominal tissue of samples after the dissection of the genitalia, using the TIANGEN™ DNA extraction kit following the recommended protocol. The *COI* fragments were amplified using the cycle protocol as in Zhao et al. (2009). The PCR sequencing primer pair was 5′–CGCCTAAACTTCAGCCACTT–3′ (Wang et al. 2006) and 5′–TAAACTTCAGGGTGACCAAAAAATCA–3′ (Folmer et al. 1994). All sequences generated in this study were supplied with BOLD Process ID and GenBank accession numbers (Table 1).

**Table 1.** Specimens of *brunnea* species used for molecular study.

	BOLD Process ID	GenBank accession number	Collection sites
*S. puncticeps* –1	BDORM010–14	KJ841771	Shennongjia, Hubei
*S. puncticeps* –2	BDORM011–14	KJ841770	Danba, Ganzizhou, Sichuan
*S. puncticeps* –3	BDORM012–14	KJ841766	Tianmushan, Linan, Zhejiang
*S. puncticeps* –4	BDORM013–14	KJ841769	Daozhen, Zunyi, Guizhou
*S. puncticeps* –5	BDORM014–14	KJ841768	Ailaoshan, Jingdong, Yunnan
*S. puncticeps* –7	BDORM004–14	KJ841761	Shennongjia, Hubei
*S. puncticeps* –8	BDORM005–14	KJ841762	Miyaluo, Abazhou, Sichuan
*S. riverata* –1	BDORM008–14	KJ841773	Banli, Chongzuo, Guangxi
*S. riverata* –2	BDORM009–14	KJ841772	Ailaoshan, Jingdong, Yunnan
*S. riverata* –3	SDLY001–19	MK335597	Likan, Ximeng, Yunnan
*S. riverata* –4	SDLY002–19	MK335598	Likan, Ximeng, Yunnan
*S. riverata* –5	SDLY003–19	MK335599	Likan, Ximeng, Yunnan
*S. riverata* –6	SDLY004–19	MK335600	Huanglianshan, Lvchun, Yunnan
*S. riverata* –7	SDLY005–19	MK335601	Huanglianshan, Lvchun, Yunnan
*S. abdentata* sp. nov. –1	BDORM019–14	KJ841758	Nanling, Shaoguan, Guangdong
*S. abdentata* sp. nov. –2	BDORM020–14	KJ841757	Muotuo, Lingzhi, Xizang
*S. abdentata* sp. nov.–3	BDORM021–14	KJ841755	Wangtianshu, Mengla, Yunnan
*S. abdentata* sp. nov. –4	SDLY006–19	MK335586	Muyiji, Ximeng, Yunnan
*S. abdentata* sp. nov. –5	BDORM023–14	KJ841759	Wuzhishan, Ledong, Hainan
*S. abdentata* sp. nov. –6	BDORM024–14	KJ841756	Muotuo, Lingzhi, Xizang
*S. abdentata* sp. nov. –7	SDLY007–19	MK335587	Muyiji, Ximeng, Yunnan
*S. abdentata* sp. nov. –8	SDLY008–19	MK335588	Mengdong, Cangyuan, Yunnan
*S. abdentata* sp. nov. –9	SDLY009–19	MK335589	Mengdong, Cangyuan, Yunnan
*S. platyrhina* sp. nov. –1	BDORM016–14	KJ841765	Menglun, Mengla, Yunnan
*S. platyrhina* sp. nov. –2	BDORM017–14	KJ841764	Menglun, Mengla, Yunnan
*S. platyrhina* sp. nov. –3	BDORM018–14	KJ841763	Jiangcheng, Simao, Yunnan
*S. platyrhina* sp. nov. –4	SDLY010–19	MK335590	Menglun, Mengla, Yunnan
*S. platyrhina* sp. nov. –5	SDLY011–19	MK335591	Guanleigang, Mengla, Yunnan
*S. platyrhina*sp. nov. –6	SDLY012–19	MK335592	Menglun, Mengla, Yunnan
*S. platyrhina* sp. nov. –7	SDLY013–19	MK335593	Menglun, Mengla, Yunnan
*S. platyrhina* sp. nov. –8	SDLY014–19	MK335594	Menglun, Mengla, Yunnan
*S. platyrhina* sp. nov. –9	SDLY015–19	MK335595	Menglun, Mengla, Yunnan
*S. platyrhina* sp. nov. –10	SDLY016–19	MK335596	Menglun, Mengla, Yunnan
*S. serrateifoliacea* sp. nov. –1	BDORM006–14	KJ841775	Hesong, Menghai, Yunnan
*S. serrateifoliacea* sp. nov. –2	BDORM007–14	KJ841774	Hesong, Menghai, Yunnan
*S. serrateifoliacea* sp. nov. –3	SDLY017–19	MK335602	Mengdong, Cangyuan, Yunnan
*S. sinuata* sp.nov. –1	SDLY024–19	MK335603	Dayangcha, Kuangdian, Liaoning
*S. sinuata* sp.nov. –2	SDLY025–19	MK335604	Dayangcha, Kuangdian, Liaoning
*S. sinuata* sp.nov. –3	SDLY026–19	MK335605	Laobiangou, Benxi, liaoning
*S. sinuata* sp.nov. –4	SDLY027–19	MK335606	Laobiangou, Benxi, liaoning
*S. sinuata* sp.nov. –5	SDLY028–19	MK335607	Laobiangou, Benxi, liaoning
*S. sinuata* sp.nov. –6	SDLY029–19	MK335608	Guojiapuzi, Kuangdian, Liaoning
*S. sinuata* sp.nov. –7	SDLY030–19	MK335609	Guojiapuzi, Kuangdian, Liaoning
*S. sinuata* sp.nov. –8	SDLY031–19	MK335610	Guojiapuzi, Kuangdian, Liaoning
*S. tanyrhina* sp. nov. –1	SDLY018–19	MK335611	Menglun, Mengla, Yunnan
*S. tanyrhina* sp. nov. –2	SDLY019–19	MK335612	Menglun, Mengla, Yunnan
*S. tanyrhina* sp. nov. –3	SDLY020–19	MK335613	Wangtianshu, Mengla, Yunnan
*S. tanyrhina* sp. nov. –4	SDLY021–19	MK335614	Wangtianshu, Mengla, Yunnan
*S. tanyrhina* sp. nov. –5	SDLY022–19	MK335615	Guanleigang, Mengla, Yunnan
*S. tanyrhina* sp. nov. –6	SDLY023–19	MK335616	Wangtianshu, Mengla, Yunnan

A total of 51 *COI* sequences of the *riverata* group were examined and aligned with MEGA 7.0 (Kumar et al. 2016). Intra- and interspecific genetic distances were calculated for species of the *riverata* group using the *p*–distance model (Nei and Kumar 2000). We also conducted character-based species barcoding where fixed sites of one species differed from those of the others; these were manually selected as diagnostic sites (i.e., “pure” diagnostics, Sarkar et al. 2002; Desalle et al. 2005). Three known species: *S. melanogaster* (GenBank accession number: KR070823), *S. rhina* (KR070845) and *S. scutellimargo* (KR070847), were used as outgroup taxa in the phylogenetic analyses. The alignment was subsequently employed to reconstruct a phylogenetic tree using the Neighbor-joining (NJ) method with *p*-distance model implemented in MEGA 7.0.26 (Kumar et al. 2016). Nodal support values (bootstrap percentages, BPs) were inferred by bootstrapping with 1000 replicates and other default settings.

### Description of species

An Mshot Camera was used to photomicrograph all the examined species. All photographs, illustrations, and line drawings were processed with the software Adobe Photoshop 7.0 and Easy PaintTool SAI Ver.1.0.0. The morphological terminology follows McAlpine (1981) and the definitions of measurements, indices, and abbreviations follow Chen & Toda (2001).

The type specimens were deposited in Department of Entomology, South China Agricultural University, Guangzhou, China (SCAU).

## Results

The alignment of the 51 *COI* sequences spans 632 nucleotide sites, with 131 variable sites, 122 of which were parsimony informative. Intra- and interspecific *p*-distances were provided in Table 2. The results show that the largest intraspecific *p*-distances within the *riverata* species group was 0.032 detected in *S. puncticeps*, followed by 0.016 in *S. platyrhina* sp. nov. while the minimum interspecific variation was 0.014 detected between *S. abdentata* sp. nov. and *S. tanyrhina* sp. nov.

**Figure 1.**
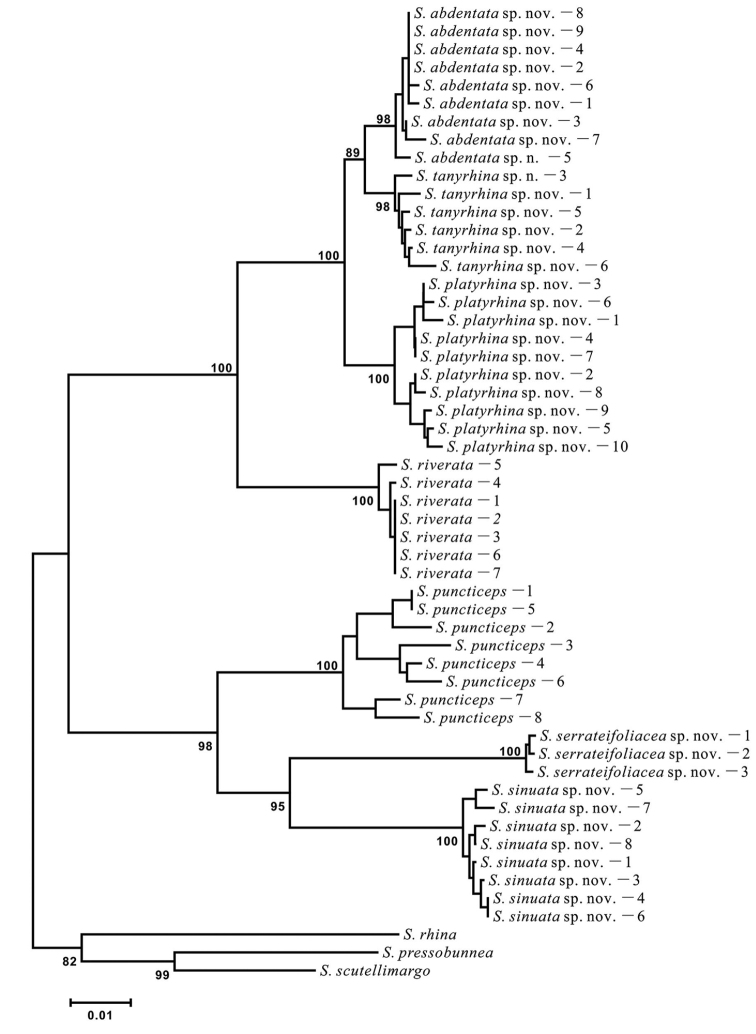
Neighbor-joining (NJ) tree of the *riverata* group. The numbers around the nodes are bootstrap (BP) percentages. BP values lower than 50 are not shown.

**Figure 2.**
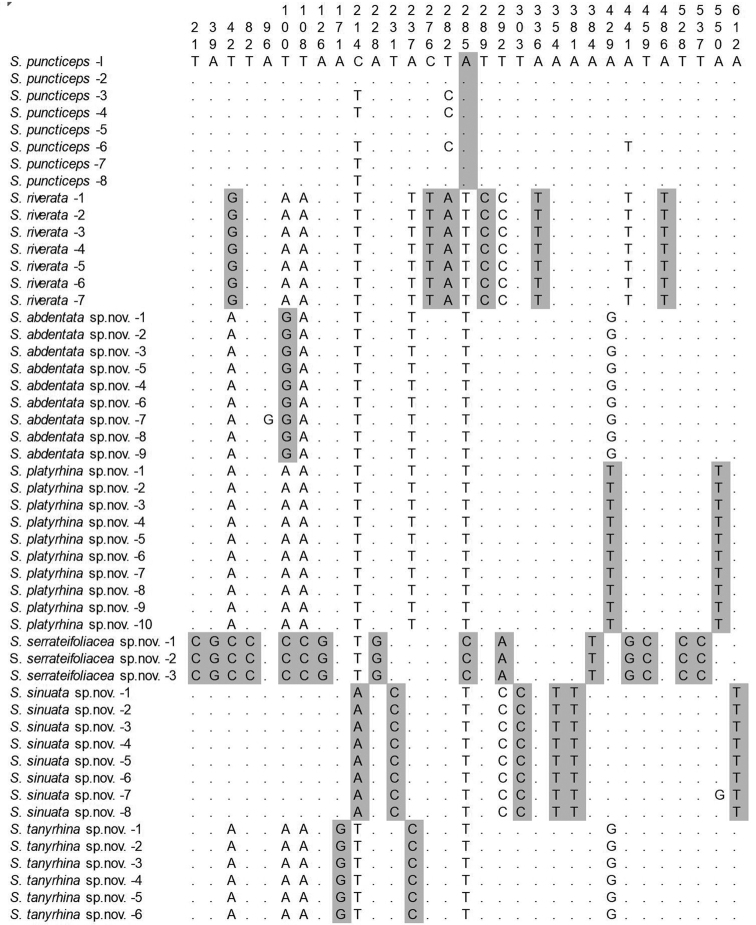
Diagnostic nucleotide sites in the alignment of *COI* sequences of the *riverata* species group. Numbers at the top show the positions of the sites in the *COI* alignment (584–687 bp in length). Shaded sites are diagnostic for each species. Dashes (–) indicate missing data.

The NJ (Fig. 1) tree shows that this new group is monophyletic with respect to the outgroups. Figure 2 shows nucleotides representing “pure” diagnostic sites for all species of the *riverata* group; at least one diagnostic site was recognized for each species. For example, site 21 is diagnostic for *S. serrateifoliacea* sp. nov. with a fixed status of C (Cytosine), rather than T (Thymidine) in the other species.

**Table 2.** Summary of intra- and interspecific genetic distances.

Species	N	intraspecific genetic distances	interspecific genetic distances
		Min. /Max. /Mean ± SD	Min. /Max. /Mean ± SD
*S. puncticeps*	8	0.000/ 0.032/0.020 ± 0.008	0.071/0.127/0.107 ± 0.017
*S. riverata*	7	0.000/ 0.006/0.002 ± 0.002	0.049/0.138/0.084 ± 0.032
*S. abdentata*	9	0.000/ 0.006/0.003 ± 0.002	0.014/0.136/0.073 ± 0.048
*S. platyrhina*	10	0.000/ 0.016/0.007 ± 0.004	0.022/0.136/0.078 ± 0.046
*S. serrateifoliacea*	3	0.002/ 0.003/0.002 ± 0.001	0.070/0.138/0.116 ± 0.026
*S. sinuata*	8	0.000/0.009/0.005 ± 0.003	0.070/0.134/0.113 ± 0.023
*S. tanyrhina*	6	0.003/ 0.009/0.005 ± 0.002	0.014/0.134/0.070 ± 0.048

N, the numbers of *COI* sequences involved in distance calculation; Min., minimum; Max., maximum; SD, standard deviation; NA, no applicable.

### Taxonomy

#### Scaptodrosophila riverata

Taxon classificationAnimaliaDipteraDrosophilidae

species group

61434C05-2AA8-54C7-92E9-4C06083CFB11

##### Diagnosis.

Body mostly yellow to yellowish brown, without patches; arista with two dorsal branches and one ventral branch in addition to the terminal bifurcation; facial carina large; prescutellar setae usually large, as long as anterior dorsocentral setae; hypandrium usually with a pair of long paramedian setae.

##### Description.

**Figure 3.**
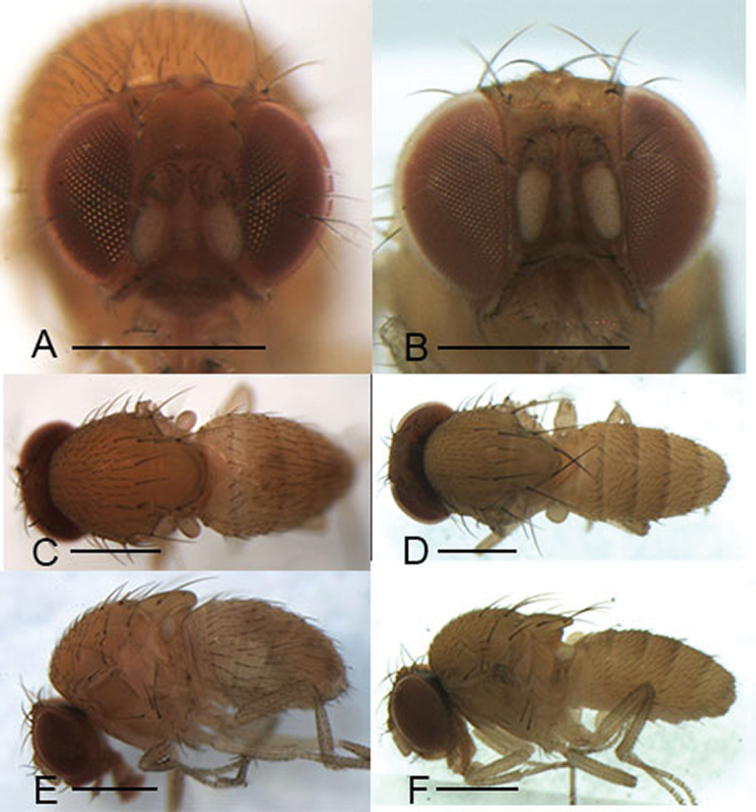
Head, mesonotum, scutellum, pleura and abdomen of male. **A, C, E***S. riverata* (Singh & Gupta, 1977) **B, D, F***S. puncticeps* (Okada, 1956). Scale bars: 0.5 mm.

**Figure 4.**
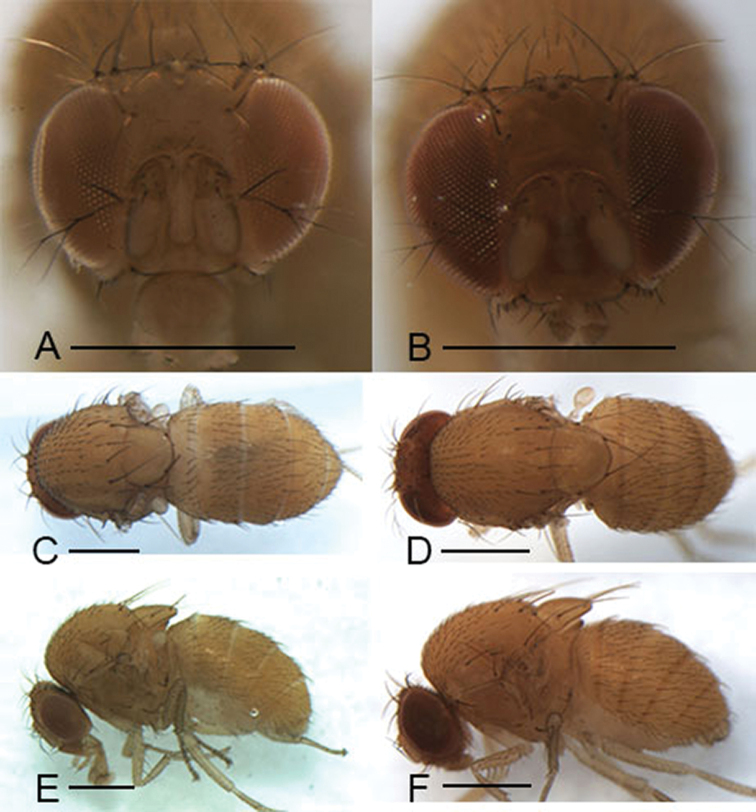
Head, mesonotum, scutellum, pleura and abdomen of male. **A, C, E***S. abdentata* sp. nov. **B, D, F***S. platyrhina* sp. nov. Scale bars: 0.5 mm.

**Figure 5.**
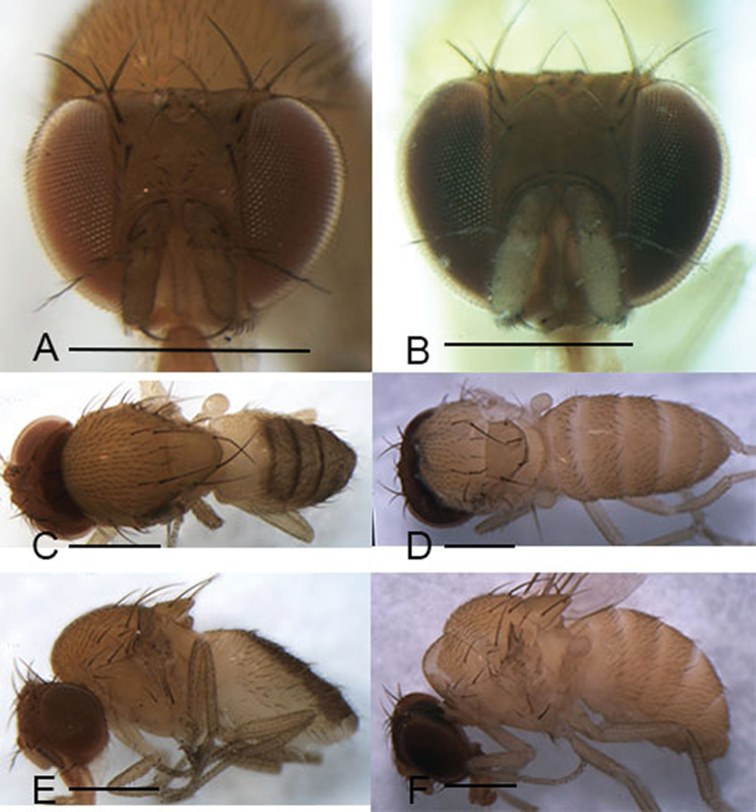
Head, mesonotum, scutellum, pleura and abdomen of male. **A, C, E***S. serrateifoliacea* sp. nov. **B, D, F***S. sinuata* sp. nov. Scale bars: 0.5 mm.

**Figure 6.**
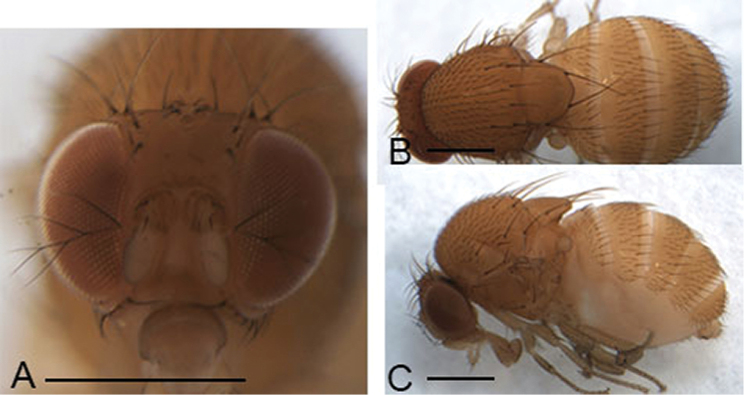
Head, mesonotum, scutellum, pleura and abdomen of male. **A–C***S. tanyrhina* sp. nov. Scale bars: 0.5 mm.

Male and female: ***Head*** (Figs 3, 4, 5A, B, 6A): Eyes red. Ocellar triangle yellowish brown, with 4–6 setae above ocellar setae. Frons about 1/3 width of head, largely yellowish brown to brown, with a few minute setulae medially. Fronto–orbital pale yellowish brown; anterior reclinate orbital seta usually lateral to and about 1/3 length of proclinate orbital seta; posterior reclinate orbital seta larger than other two orbital setae. Pedicel yellowish brown to brown, with a few of fine setae; first flagellomere pale yellowish. Face yellowish brown. Clypeus mostly yellow. Vibrissa prominent; subvibrissal setae small. Gena and postgena narrow, yellowish brown. Palpus yellow to yellowish brown with several setae.

***Thorax*** (Figs 3, 4, 5C–F, 6B, C): Mesonotum yellow to yellowish brown uniformly. Postpronotal lobe yellow with 2–3 long setae, and a few of shorter setae. Acrostichal setulae in ca 8–10 regular rows. Prescutellar setae usually as long as anterior dorsocentral setae. Pleura yellow to yellowish brown uniformly. Katepisternal setae usually subequal. Wing hyaline, sometimes infuscate, lacking patch. Basal medial-cubital crossvein absent. R_4+5_ nearly parallel distally with M_1_. Halter pale yellowish. Legs mostly yellow.

***Abdomen*** (Figs 3, 4, 5C–F, 6B, C): Tergites yellow to yellowish brown, lacking patches.

**Figure 7.**
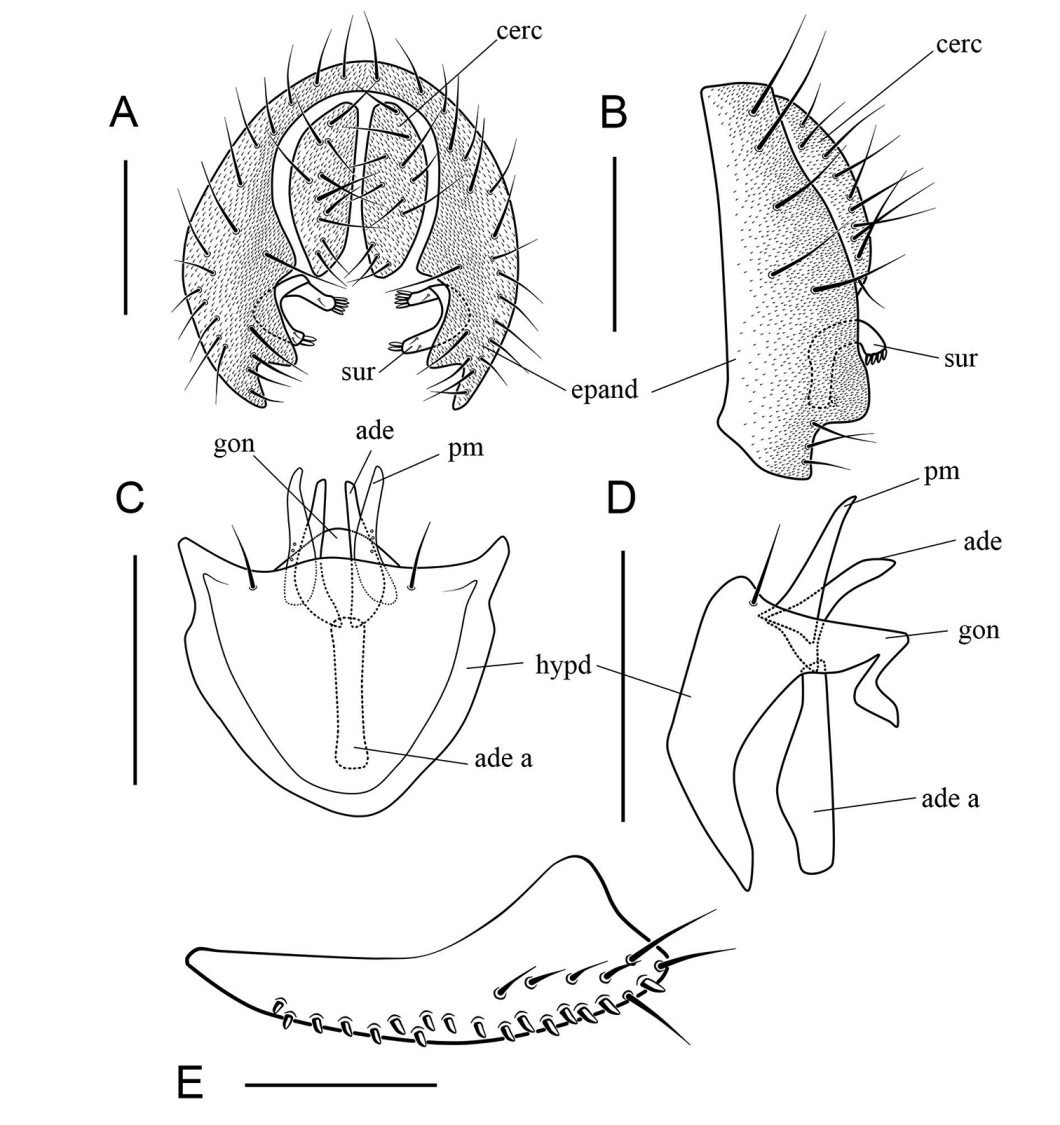
*Scaptodrosophila riverata* (Singh & Gupta, 1977) ♂. **A** Epandrium, surstylus and cercus (posterior view) **B** surstylus (lateral view) **C** hypandrium, aedeagus, aedeagal apodeme, paramere and gonopods (ventral view) **D** hypandrium, aedeagus, aedeagal apodeme, paramere and gonopods (lateral view) **E** oviscapt (lateral view). Scale bars: 0.1 mm.

**Figure 8.**
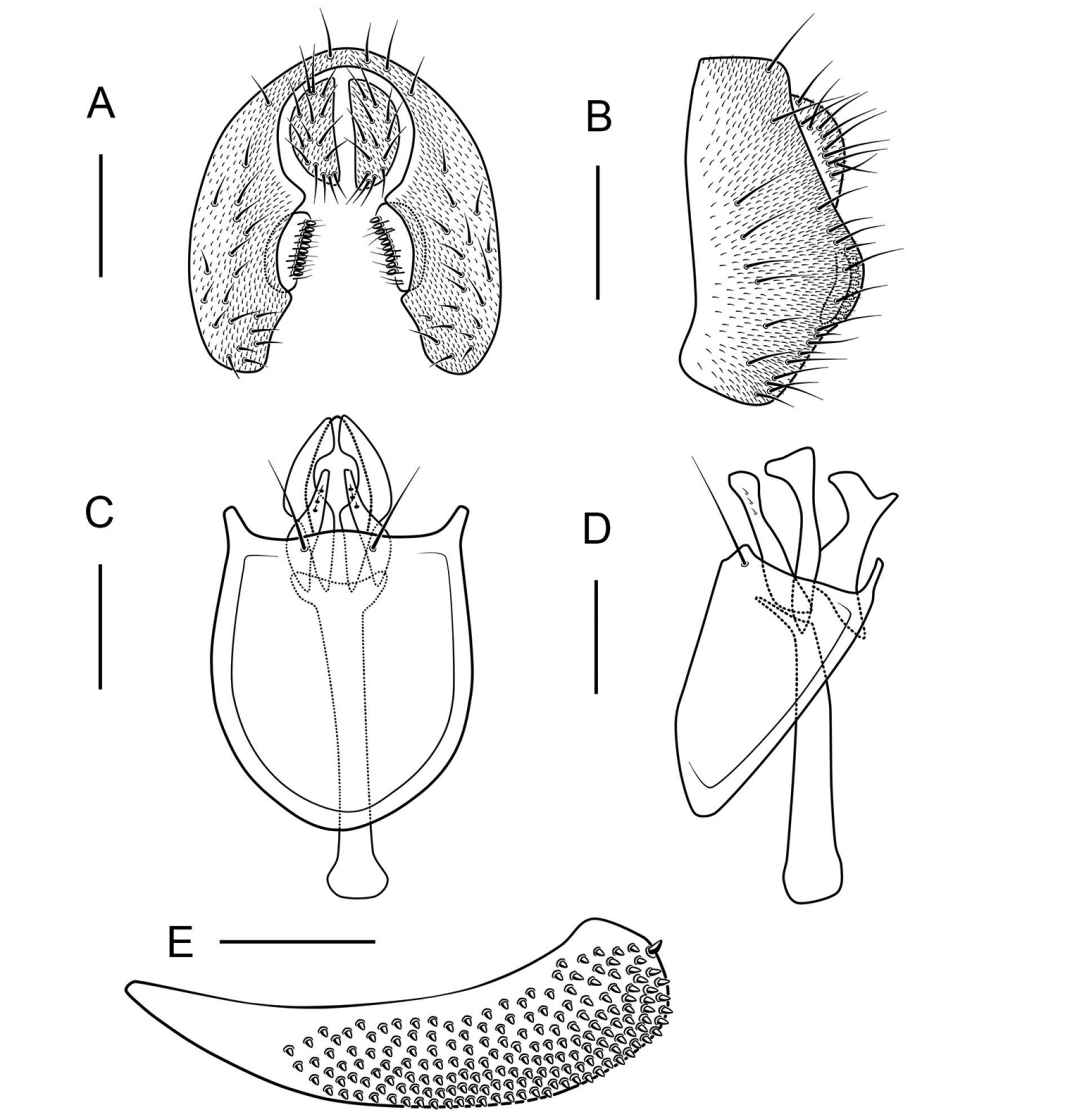
*Scaptodrosophila puncticeps* (Okada, 1956) ♂. **A** Epandrium, surstylus and cercus (posterior view) **B** epandrium, surstylus and cercus (lateral view) **C** hypandrium, aedeagus, aedeagal apodeme, paramere and gonopods (ventral view) **D** hypandrium, aedeagus, aedeagal apodeme, paramere and gonopods (lateral view) **E** oviscapt (lateral view). Scale bars: 0.1 mm.

**Figure 9.**
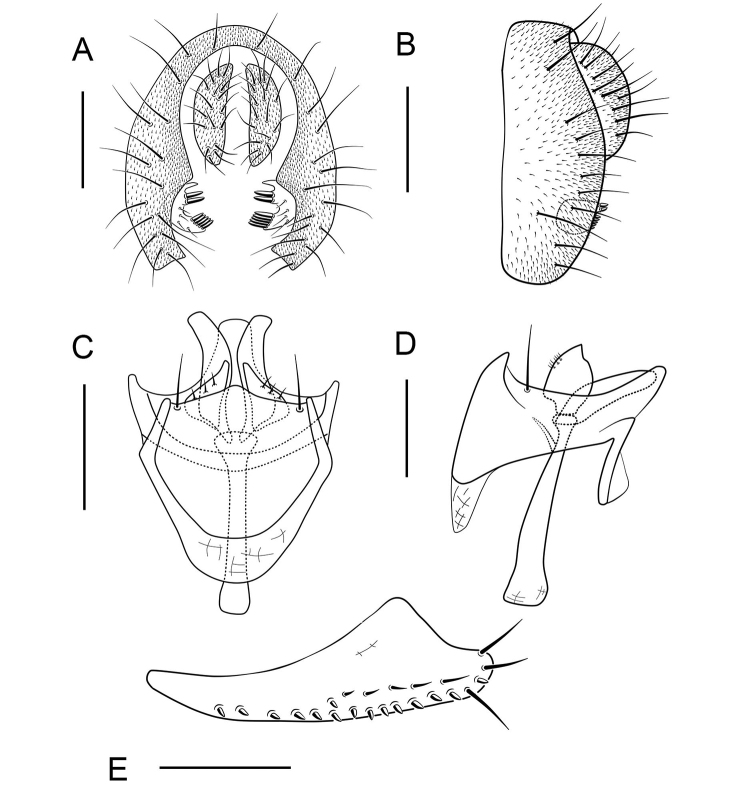
*Scaptodrosophila abdentata* sp. nov. ♂. **A** Epandrium, surstylus and cercus (posterior view) **B** epandrium, surstylus and cercus (lateral view) **C** hypandrium, aedeagus, aedeagal apodeme, paramere and gonopods (ventral view) **D** hypandrium, aedeagu, aedeagal apodeme, paramere and gonopods (lateral view) **E** oviscapt (lateral view). Scale bars: 0.1 mm.

**Figure 10.**
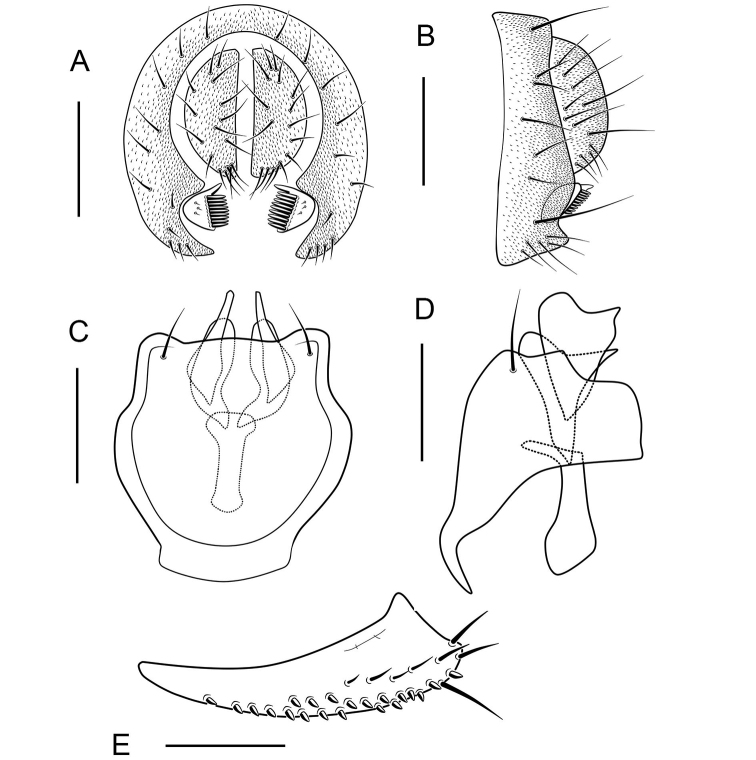
*Scaptodrosophila platyrhina* sp. nov. ♂. **A** Epandrium, surstylus and cercus (posterior view) **B** epandrium, surstylus and cercus (lateral view) **C** hypandrium, aedeagus, aedeagal apodeme, paramere and gonopods (ventral view) **D** hypandrium, aedeagus, aedeagal apodeme, paramere and gonopods (lateral view) **E** oviscapt (lateral view). Scale bars: 0.1 mm.

**Figure 11.**
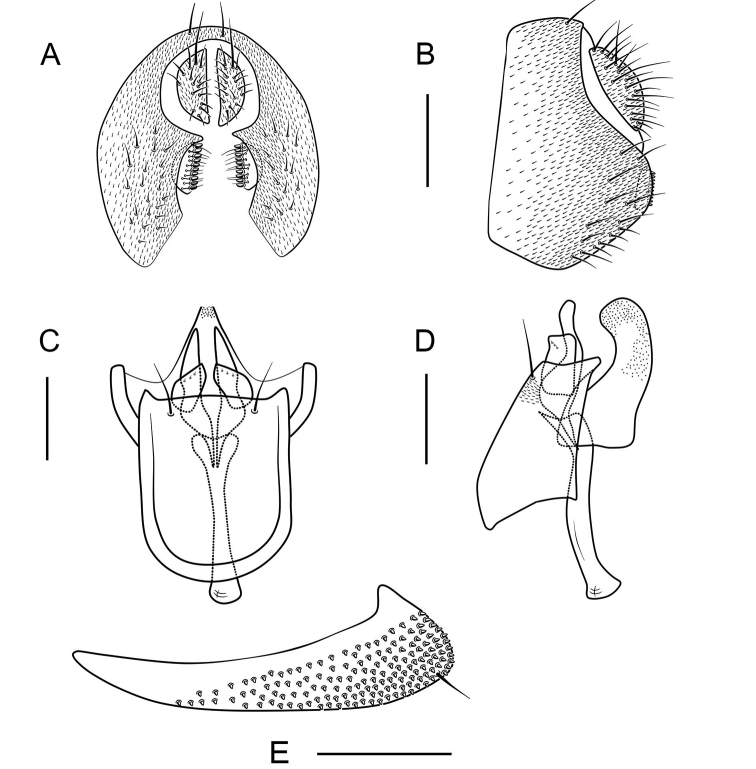
*Scaptodrosophila serrateifoliacea* sp. nov. ♂. **A** Epandrium, surstylus and cercus (posterior view) **B** epandrium, surstylus and cercus (lateral view) **C** hypandrium, aedeagus, aedeagal apodeme, paramere and gonopods (ventral view) **D** hypandrium, aedeagus, aedeagal apodeme, paramere and gonopods (lateral view) **E** oviscapt (lateral view). Scale bars: 0.1 mm.

**Figure 12.**
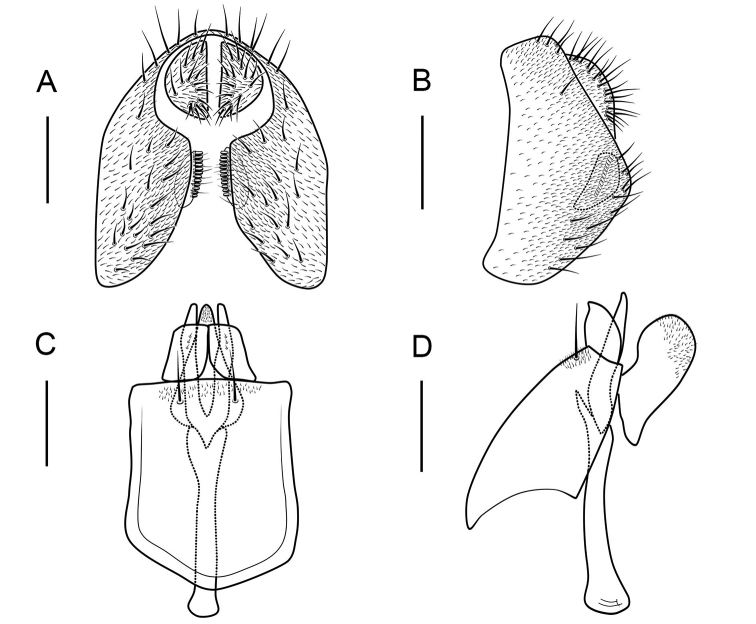
*Scaptodrosophila sinuata* sp. nov. ♂. **A** Epandrium, surstylus and cercus (posterior view) **B** epandrium, surstylus and cercus (lateral view) **C** hypandrium, aedeagus, aedeagal apodeme, paramere and gonopods (ventral view) **D** hypandrium, aedeagus, aedeagal apodeme, paramere and gonopods (lateral view). Scale bars: 0.1 mm.

**Figure 13.**
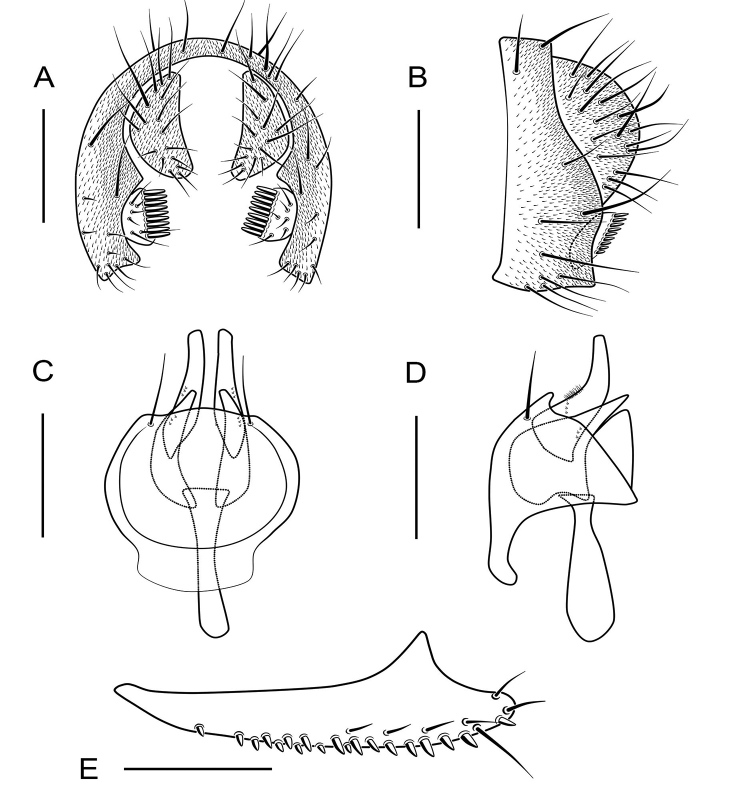
*Scaptodrosophila tanyrhina* sp. nov. ♂. **A** Epandrium, surstylus and cercus (posterior view) **B** epandrium, surstylus and cercus (lateral view) **C** hypandrium, aedeagus, aedeagal apodeme, paramere and gonopods (ventral view) **D** hypandrium, aedeagus, aedeagal apodeme, paramere and gonopods (lateral view) **E** oviscapt (lateral view). Scale bars: 0.1 mm.

***Male terminalia*** (Figs 7–12, 13A–D): Epandrium pubescent, with several setae near posterior margin and ventral corner on each side. Surstylus usually with several peg-like prensisetae on caudal margin, and several setae on outer and inner surface. Hypandrium shallow, usually with a couple of paramedian setae distally. Cercus separated from epandrium, pubescent and setigerous. Paramere developed, with few sensillae. Gonopods (as dorsal extension of the hypandrium, see Ashley and Sinclair 2017) fused with each other, broadened to hood-shaped. Aedeagus bifid, glabrous.

***Female terminalia*** (Figs 7–10, 11E, 13E): Oviscapt mostly yellowish brown to brown, broadened subapically.

In the following descriptions, only those characters differing from the above description were provided.

#### Scaptodrosophila riverata

Taxon classificationAnimaliaDipteraDrosophilidae

(Singh & Gupta, 1977)

505B9084-E667-5A69-92C7-1C66E50C32D5

Figs 3A, C, E , 7

Drosophila riverata Singh & Gupta, 1977: 240.

##### Material examined.

China: 2♂ (SCAU, Nos 126828, 29), Banli, Chongzuo, Guangxi, alt. 160 m, 21.viii.2004, HW Chen; 1♀ (SCAU, No. 126830), Ailaoshan, Jingdong, Yunnan, 24°32'N, 101°00'E, alt. 2100 m, 21.vi.2009, T Li; 3♂ (SCAU, Nos 110913–15), Likan, Ximeng, Yunnan, 22°39'N, 99°36'E, alt. 844 m, 1.v.2016, YQ Liu; 1♂, 1♀ (SCAU, Nos 110916, 17), Huanglianshan, Lüchun, Yunnan, 23°20'N, 102°23'E, alt. 600 m, 28.x.2018, HW Chen.

##### Diagnosis.

Surstylus bifid, the upper lobe with about 5 thin, peg-like prensisetae and 1 fine seta, the below one with 1 thin, peg-like prensiseta and 2 fine setae (Fig. 7A, B); gonopods undeveloped (Fig. 7C, D).

##### Description.

(♂, ♀) ***Head*** (Fig. 3A): Frons yellowish brown. Pedicel and first flagellomere maple. Facial carina yellowish, short, 1/3 length as face.

***Thorax*** (Fig. 3C, E): Mesonotum and scutellum yellowish. Acrostichal setulae in ca 8 regular rows. Wings hyaline.

***Abdomen*** (Fig. 3C, E): Tergites and sternites yellowish.

***Male terminalia***: Epandrium with ca 14 setae near posterior margin and ventral corner on each side (Fig. 7A, B). Hypandrium glabrous (Fig. 7C, D). Paramere slender and curved apically, with 3 sensillae subbasally (Fig. 7C, D).

***Female terminalia*** (Fig. 7E): Oviscapt with 2 long subapical trichoid ovisensilla, 17 peg-like ovisensilla on each side on ventral margins and 5 trichoid ovisensilla on dorsal.

***Measurements*** (range in 2♂, 1♀, in mm): BL = (1.73–2.07, 1.93 mm), ThL = (0.80–1.07, 0.80), WL = (1.80–2.27, 1.93), WW = (0.67–0.93, 0.80).

***Indices***: arb = 2/1, avd = 0.60–1.00, adf = 2.00–2.50, flw = 2.00, FW/HW = 0.45–0.48, ch/o = 0.08, prorb = 0.80–0.83, rcorb = 0.40–0.60, vb = 0.33–0.50, dcl = 0.54–0.60, presctl = 0.45–0.60, sctl = 1.00–1.10, sterno = 0.67–0.75, orbito = 0.67–0.75, dcp = 0.30–0.40, sctlp = 0.83, C = 2.42–2.92, 4c = 1.00–1.20, 4v = 2.17–2.60, 5x = 1.50–2.00, ac = 2.40–3.00, M = 0.67–0.80, C3F = 0.25–0.33.

##### Distribution.

China (Guangxi, Yunnan), India, Myanmar.

#### Scaptodrosophila puncticeps

Taxon classificationAnimaliaDipteraDrosophilidae

(Okada, 1956)

33D2C739-3A07-5E1E-9D8E-91238AA9E37C

Figs 3B, D, F , 8

Drosophila puncticep s Okada, 1956: 94.

##### Material examined.

China: 6♂, 5♀ (SCAU, Nos 126796–804, 830), Shennongjia, Hubei, 31°49'N, 109°41'E, alt. 1900 m, 31.vii. 2004, 6.viii.2005, HW Chen, HZ Cao; 1♂ (SCAU, No. 126831), Tianmushan, Linan, Zhejiang, 30°20'N, 119°25'E, alt. 800 m, 30.vi.–2.viii.2011, tussocks, ZF Shao, SJ Yan; 1♂ (SCAU, No. 126825), Miyaluo, Aba, Sichuan, alt. 2650 m, 14.ix.2005, MF Xu.

##### Diagnosis.

Clypeus reddish brown; palpus pale yellow; gonopods roundly expanded in lateral view. (Fig. 7C, D).

##### Description.

(♂, ♀) ***Head*** (Fig. 3B): Frons reddish brown. Pedicel yellowish brown. Facial carina creamy white, short, 1/3 length as face.

***Thorax*** (Fig. 3D, F): Mesonotum and scutellum yellow. Acrostichal setulae in ca 7 irregular rows. Halter yellowish brown. Wing greyish.

***Abdomen*** (Fig. 3D, F): Tergites yellowish brown.

***Male terminalia***: Epandrium with ca 21 setae near posterior margin and ventral corner on each side (Fig. 8A, B). Surstylus small with 10 peg-like prensisetae on caudal margin, and ca 16 setae on outer and inner surface (Fig. 8A, B). Hypandrium pubescent near paramedian setae (Fig. 8C, D). Paramere broadened medially with ca 3 sensilla (Fig. 8C, D). Aedeagus broadened apically, curved medially, sickle-shaped anteriorly in lateral view (Fig. 8C, D).

***Female terminalia*** (Fig. 8E): Oviscapt densely covered with peg-like ovisensilla.

***Measurements*** (range in 6♂, 5♀, in mm): BL = 1.87–3.87, 2.20–2.67, ThL = 0.80–1.13, 0.80–1.00, WL = 2.13–2.93, 2.13–2.73, WW = 0.73–1.20, 0.86–1.07.

***Indices***: arb = 2/1, avd = 0.25–1.00, adf = 1.50–2.00, flw = 2.00–4.00, FW/HW = 0.41–0.50, ch/o = 0.07–0.13, prorb = 0.71–1.00, rcorb = 0.29–0.50, vb = 0.33–0.75, dcl = 0.45–0.70, presctl = 0.33–0.57, sctl = 0.85–1.22, sterno = 0.30–0.67, orbito = 0.50–0.75, dcp = 0.44–0.56, sctlp = 0.71–1.25, C = 2.75–4.00, 4c = 0.60–0.92, 4v = 1.40–2.08, 5x = 1.20–1.67, ac = 0.86–2.33, M = 0.40–0.60, C3F = 0.30–0.38.

##### Distribution.

China (Zhejiang, Hubei, Hunan, Sichuan, Guizhou, Yunnan), Kuril Islands, Korea, Japan (Kanto).

#### Scaptodrosophila abdentata sp. nov.

Taxon classificationAnimaliaDipteraDrosophilidae

221BABE6-EF36-54F8-8234-2BC846666D96

http://zoobank.org/426657F1-40A2-4E11-BFB1-BBCB8B7A2D42

Figs 4A, C, E , 9

##### Material examined.

***Holotype.*** China: ♂ (SCAU, No. 127162), Nanling, Shaoguan, Guangdong, 24°38'N, 112°40'E, alt. 800 m, 3.iii.2004, MF Xu. ***Paratypes.*** China: 1♂, 2♀ (SCAU, Nos 127163–65), Nanling, Ruyuan, Guangdong, 24°38'N, 112°40'E, alt. 800 m, 3.iii.2004, MF Xu; 1♂, 2♀ (SCAU, Nos 127166–68), Jianfengling, Ledong, Hainan, 18°41'N, 108°52'E, alt. 700 m, 23.iv.2007, HW Chen; 1♂, 1♀ (SCAU, Nos 127169–70), Beibeng, Motuo, Xizang, 29°19'N, 95°20'E, alt. 780 m, 4.x.2010, YR Su; 2♂ (SCAU, Nos 127171–72), Wangtianshu, Mengla, Yunnan, 21°28'N, 101°38'E, alt. 580 m, 23.iv.2007, HW Chen; 1♂ (SCAU, No. 110918), Muyiji, Ximeng, Yunnan, 22°37'N, 99°35'E, alt. 1100 m, 23.iv.2007, HW Chen; 1♂ (SCAU, No. 110919), Muyiji, Ximeng, Yunnan, 22°37'N, 99°35'E, alt. 1203 m, 30.iv. 2016, YQ Liu; 2♂ (SCAU, Nos 110920, 21), Mengdong, Cangyuan, Yunnan, 23°10'N, 99°13'E, alt. 1323 m, 6.v.2016, YQ Liu.

##### Diagnosis.

Surstylus with a row of ca 9 long, peg-like prensisetae on caudal margin, and ca 6 setae on outer surface (Fig. 9A, B); paramere leaf-shaped in lateral view, with ca 3 sensillae and a row of fine setae (Fig. 9C, D); aedeagus broadened apically (Fig. 9C, D).

##### Description.

(♂, ♀) ***Head*** (Fig. 4A): Frons yellowish brown. Pedicel brown. First flagellomere yellowish. Facial carina brownish, short, 1/3 length as face.

***Thorax*** (Fig. 4C, E): Mesonotum and scutellum yellowish brown. Acrostichal setulae in ca 8 regular rows. Halter hazel. Wings yellowish.

***Abdomen*** (Fig. 4C, E): Tergites creamy white.

***Male terminalia***: Epandrium with ca 15 setae near posterior margin and ventral corner on each side (Fig. 9A, B). Hypandrium glabrous (Fig. 9C, D).

***Female terminalia*** (Fig. 8E): Oviscapt with 3 long subapical trichoid ovisensilla, 15 peg-like ovisensilla on each side on ventral margins and 5 trichoid ovisensilla on dorsal.

***Measurements*** [holotype ♂ (range in 5♂, 5♀), in mm]: BL = 2.20 (1.73–1.93, 2.00–2.27), ThL = 1.07 (0.80–1.00, 1.00–1.13), WL = 2.40 (1.87–2.20, 2.20–2.33), WW = 0.93 (0.67–0.87, 0.87–0.93).

***Indices***: arb = 2/1 (2/1), avd = 0.60 (0.60–1.00), adf = 2.50 (2.00–2.50), flw = 2.00 (1.50–2.50), FW/HW = 0.39 (0.41–0.50), ch/o = 0.07 (0.08–0.10), prorb = 0.83 (0.71–1.00), rcorb = 0.50 (0.33–0.50), vb = 0.50 (0.50–0.67), dcl = 0.58 (0.54–0.75), presctl = 0.50 (0.38–0.55), sctl = 1.25 (1.00–1.31), sterno = 0.78 (0.60–0.72), orbito = 0.67 (0.67), dcp = 0.33 (0.31–0.44), sctlp = 1.83 (0.80–1.00), C = 3.23 (2.21–3.00), 4c = 0.93 (0.92–1.20), 4v =2.43 (2.21–3.00), 5x = 1.60 (1.60–2.00), ac = 2.60 (2.40–3.00), M = 0.57 (0.57–0.82), C3F = 0.38 (0.31–0.38).

##### Etymology.

A combination of the Latin words: “*ab*–” + “*dentatus*”, referring to the surstylus with a line discontinuous of prensisetae.

##### Distribution.

China (Guangdong, Hainan, Yunnan, Xizang).

#### Scaptodrosophila platyrhina sp. nov.

Taxon classificationAnimaliaDipteraDrosophilidae

F3389E7A-5499-5307-B15A-6D3714F13D7D

http://zoobank.org/E1397E1F-B748-41DE-B651-B8727E27AD34

Figs 4B, D, F , 10

##### Material examined.

***Holotype.*** China: ♂ (SCAU, No. 126838), Menglun, Mengla, Yunnan, 21°41'N, 101°25'E, alt. 680 m, 17.iv. 2007, HW Chen. ***Paratypes.*** China: 4♂, 7♀ (SCAU, Nos 126839–47), Menglun, Mengla, Yunnan, 21°41'N, 101°25'E, alt. 680 m, 17.iv.2007, HW Chen; 1♂ (SCAU, No. 127161), Niuluohe, Jiangcheng, Yunnan, alt. 800 m, 22°30'N, 101°34'E, 21.iv.2010, HW Chen; 1♂ (SCAU, No. 110923), Guanlei, Mengla, Yunnan, 21°38'N, 101°10'E, alt. 620 m, 21.iv.2016, YQ Liu; 2♂, 2♀ (SCAU, Nos 110924, 26–28), Menglun, Mengla, Yunnan, 24°41'N, 101°25'E, alt. 554 m, 12.iv.2010, HW Chen.

##### Diagnosis.

Facial carina short; paramere finger-like in lateral view, with ca 4 sensillae basally (Fig. 10C, D); aedeagus bifid apically (Fig. 10C, D); gonopods undeveloped (Fig. 10C, D).

##### Description.

***Head*** (Fig. 4B): Frons yellowish brown. Pedicel yellowish brown. First flagellomere yellowish. Facial carina hazel, short, 1/3 length as face.

***Thorax*** (Fig. 4D, F): Mesonotum and scutellum yellowish. Acrostichal setulae in ca 8 regular rows. Halter hazel. Wings yellowish.

***Abdomen*** (Fig. 4D, F): Tergites yellowish.

***Male terminalia***: Epandrium with ca 11 setae near posterior margin and ventral corner per side (Fig. 10A, B). Surstylus with ca 10 long, peg-like prensisetae on caudal margin, and ca 3 setae on outer surface (Fig. 10A, B). Hypandrium glabrous (Fig. 10C, D).

***Female terminalia*** (Fig. 10E): Oviscapt with 3 long subapical trichoid ovisensilla, ca 21 peg-like ovisensilla on each side on ventral margins and 5 trichoid ovisensilla on dorsal.

***Measurements*** [holotype ♂ (range in 5♂, 5♀), in mm]: BL = 2.27 (2.00–2.33, 1.93–2.13), THL = 1.13 (0.80–1.07, 1.00–1.07), WL = 2.47 (2.20–2.40, 2.20–2.40), WW = 0.93 (0.80–0.93, 0.80–0.93).

***Indices***: arb = 2/1 (2/1), avd = 0.60 (0.60–0.80), adf = 2.50 (2.00–2.50), flw = 2.00 (2.00), FW/HW = 0.43 (0.41–0.45), ch/o = 0.08 (0.08–0.09), prorb = 0.83 (0.40–1.00), rcorb = 0.50 (0.33–0.60), vb = 0.40 (0.40–0.50), dcl =0.71 (0.50–0.73), presctl = 0.43 (0.40–0.50), sctl = 1.00 (0.85–1.27), sterno = 0.89 (0.70–0.89), orbito = 0.67 (0.67), dcp = 0.36 (0.31–0.36), sctlp = 1.00 (0.71–1.00), C = 2.86 (2.64–2.79), 4c = 1.00 (0.93–1.08), 4v = 2.36 (2.00–2.46), 5x = 1.67 (1.50–1.80), ac = 2.80 (2.80–3.00), M = 0.71 (0.64–0.71), C3F = 0.36 (0.29–0.36).

##### Etymology.

A combination of the Latin words: “*platys*” + “*rhinos*”, referring to the flat carina.

##### Distribution.

China (Yunnan).

#### Scaptodrosophila serrateifoliacea sp. nov.

Taxon classificationAnimaliaDipteraDrosophilidae

AE69324A-94EF-542C-B120-C667BC8FE824

http://zoobank.org/9DC99991-AE49-4AC7-854B-C6CBC6AC9E88

Figs 5A, C, E , 11

##### Material examined.

***Holotype.*** China: ♂ (SCAU, No. 126826), Hesong, Menghai, Yunnan, 21°49'N, 100°06'E, alt. 1900 m, 11.iv. 2011, JM Lu, YR Su, ZF Shao, SJ Yan. ***Paratypes.*** China: 1♂ (SCAU, No. 126827), Hesong, Menghai, Yunnan, 21°50'N, 100°06'E, alt. 1900 m, 11.iv.2011, JM Lu, YR Su, ZF Shao, SJ Yan. 1♂ (SCAU, No. 110929), Mengdong, Cangyuan, Yunnan, 23°10'N, 99°13'E, alt. 1323 m, 6.v.2016, YQ Liu.

##### Diagnosis.

Paramere quadrangle-shaped in lateral view, with ca 3 sensillae medially (Fig. 11C, D); gonopods developed, arm-shaped, with many finely acanthoid processes (Fig. 11C, D); aedeagus beanpod-shaped in lateral view (Fig. 11C, D).

##### Description.

(♂, ♀) ***Head*** (Fig. 5A): Frons yellowish brown. Pedicel yellow. First flagellomere yellowish. Facial carina yellowish, short, 1/2 length as face.

***Thorax*** (Fig. 5C, E): Mesonotum and scutellum dark brown. Acrostichal setulae in ca 8 irregular rows. Halter yellowish brown. Wings hazel.

***Abdomen*** (Fig. 5C, E): First tergite and second tergite yellow. Third to sixth tergites brown.

***Male terminalia***: Epandrium with ca 19 setae near posterior margin and ventral corner on each side (Fig. 11A, B). Surstylus broad, with ca 10 fine peg-like prensisetae on caudal margin, and numerous setae on outer and inner surfaces (Fig. 11A, B). Hypandrium pubescent near paramedian setae (Fig. 11C, D).

***Female terminalia*** (Fig. 11E): Oviscapt with 1 long subapical trichoid ovisensillum on ventral margins and densely covered with peg-like ovisensilla.

***Measurements*** [holotype ♂ (range in 1♂, 1♀), in mm]: BL = 1.93 (2.22, 1.80), ThL = 0.93 (0.889, 1.00), WL = 2.13 (2.44, 2.13), WW = 0.87 (0.978, 0.93).

***Indices***: arb = 2/1 (2/1), avd = 0.67 (0.67–0.73), adf = 1.00 (1.09–1.50), flw = 1.33 (1.59–2.50), FW/HW = 0.45 (0.344–0.48), ch/o = 0.15 (0.11–0.13), prorb = 1.00 (0.71–1.40), rcorb = 0.40 (0.20–0.43), vb = 0.50 (0.59–0.67), dcl =0.50 (0.59–0.64), presctl = 0.33 (0.36–0.37), sctl = 0.92 (1.22–1.08), sterno = 0.50 (0.33–0.45), orbito = 0.67 (0.65–0.67), dcp = 0.44 (0.42–0.44), sctlp = 0.83 (1.20–1.25), C = 2.92 (2.93–4.30), 4c = 0.81 (0.55–0.82), 4v = 1.88 (1.66–1.76), 5x = 1.75 (0.94–1.33), ac = 2.60 (1.59–2.33), M = 0.44 (0.34–0.47), C3F = 0.38 (0.50–0.60).

##### Etymology.

A combination of the Latin words “*serratus*” (= serrated) + “*foliaceus*” (= folium), referring to the gonopods with numerous finely serrated processes.

##### Distribution.

China (Yunnan).

#### Scaptodrosophila sinuata sp. nov.

Taxon classificationAnimaliaDipteraDrosophilidae

1222CE54-3689-5B2E-8D10-428C9BE486D4

http://zoobank.org/972ED4E8-7F80-4F9C-9610-B9922E4EBF4B

Figs 5B, D, F , 12

##### Material examined.

***Holotype.*** China: ♂ (SCAU, No. 110939), Laobiangou, Benxi, Liaoning, 41°04'N, 124°30'E, alt. 575 m, 21.vi.2018, Y Lin. ***Paratypes.*** China: 2♂ (SCAU, Nos 110936, 37), Dayangcha, Kuangdian, Liaoning, 41°01'N, 124°37'E. alt. 485 m, 23.vi.2018, L Gong; 2♂ (SCAU, Nos 110938, 40), Laobiangou, Benxi, Liaoning, 41°04'N, 124°30'E, alt. 575 m, 21.vi.2018, Y Lin; 3♂ (SCAU, Nos 110941, 42, 43), Guojiapuzi, Kuangdian, Liaoning, 40°46'N, 124°45'E. alt. 342 m, 26.vi.2018, L Gong.

##### Diagnosis.

Paramere quadrangle-shaped in lateral view, with ca 3 sensillae medially (Fig. 12A, B); gonopods large, with many finely acanthoid processes (Fig. 12C, D); aedeagus beanpod-shaped in lateral view (Fig. 12C, D).

##### Description.

(♂) ***Head*** (Fig. 5B): Frons yellowish brown. Pedicel yellow. First flagellomere yellowish brown. Arista weak. Facial carina yellowish, developed, 1/2 length as face.

***Thorax*** (Fig. 5D, F): Mesonotum and scutellum yellowish. Acrostichal setulae in ca 8 irregular rows. Halter yellowish brown. Wings light brown and appreciably hyaline.

***Abdomen*** (Fig. 5D, F): Tergites yellowish.

***Male terminalia*** (Fig. 12): Epandrium with ca 23 setae near posterior margin and ventral corner on each side (Fig. 12A, B). Surstylus broad, with ca 12 fine peg-like prensisetae on caudal margin, and numerous setae on outer and inner surface (Fig. 12A, B). Hypandrium pubescent near paramedian setae (Fig. 12C, D).

***Measurements*** [holotype ♂ (range in 3♂), in mm]: BL = 2.36 (2.33–2.44), ThL = 0.978 (1.02–1.07), WL = 2.33 (2.07–2.30), WW = 0.99 (0.99–1.02).

***Indices***: arb = 2/1 (2/1), avd = 0.88 (0.67–0.88), adf = 0.97 (0.97–0.99), flw = 1.56 (1.51–1.62), FW/HW = 0.36 (0.35–0.37), ch/o = 0.12(0.11–0.13), prorb = 0.82 (0.95–0.98), rcorb = 0.50 (0.50–0.52), vb = 0.73 (0.47 –0.52), dcl =0.57 (0.71–0.72), presctl = 0.36 (0.42–0.44), sctl = 1.873 (1.62–1.72), sterno = 0.43 (0.41–0.43), orbito = 0.48 (0.48–1.07), dcp = 0.41 (0.40–0.42), sctlp = 0.77 (0.76–0.82), C = 3.79 (3.76–4.09), 4c = 0.59 (0.53–0.60), 4v =1.69 (1.72–1.80), 5x = 0.86 (0.86–0.95), ac =1.36 (1.35–1.71), M = 0.31 (0.29–0.32), C3F = 0.60 (0.56–0.58).

##### Etymology.

From the Latin word: “*sinuatus*”, referring to the paramere, which is curved subbasally.

##### Distribution.

China (Liaoning).

#### Scaptodrosophila tanyrhina sp. nov.

Taxon classificationAnimaliaDipteraDrosophilidae

5BF42988-BCC1-5D08-ACB0-FDEC1CAE051B

http://zoobank.org/20E556DF-CD47-4ACD-AB29-F9C8ADCA351B

Figs 6A, B, C , 13

##### Material examined.

***Holotype.*** China: ♂ (SCAU, No. 126401), Menglun, Mengla, Yunnan, 21°41'N, 101°25'E, alt. 780 m, 17.iv.2007, HW Chen, JJ Gao. ***Paratypes.*** China: 7♂, 5♀ (SCAU, Nos 126402–11, 110930–31), Menglun, Mengla, Yunnan, 21°41'N, 101°25'E, alt. 780 m, 17.iv.2007, HW Chen, JJ Gao; 2♂, 1♀ (SCAU, Nos 110932, 33, 35), Wangtianshu, Mengla, Yunnan, 21°28'N, 101°38'E, alt. 600 m, 23.iv.2007, HW Chen; 1♂ (SCAU, No. 110934), Guanglei, Mengla, Yunnan, 21°38'N, 101°10'E, alt. 620 m, 21.iv.2016, YQ Liu.

##### Diagnosis.

Paramere broadened basally, finger-like in lateral view, with ca 6 sensillae subbasally and fine setae medially (Fig. 13C, D); gonopods undeveloped; aedeagus broadened apically, curved in lateral view (Fig. 13C, D).

##### Description.

(♂, ♀) ***Head*** (Fig. 6A): Frons yellowish brown. Pedicel yellowish brown. First flagellomere yellowish. Facial carina yellowish, short, 1/3 length as face.

***Thorax*** (Fig. 6B, C): Mesonotum and scutellum yellowish brown. Acrostichal setulae in ca 8 regular rows. Halter yellowish brown. Wings yellowish.

***Abdomen*** (Fig. 6B, C): Tergite yellowish brown. Sternites yellowish.

***Male terminalia***: Epandrium with ca 13 setae near posterior margin and ventral corner on each side (Fig. 13A, B). Surstylus small with ca 9 peg-like prensisetae on caudal margin, and ca 6 setae on outer and inner surface (Fig. 13A, B). Hypandrium glabrous (Fig. 13C, D).

***Female terminalia*** (Fig. 13E): Oviscapt with 3 long subapical trichoid ovisensilla, ca 19 peg-like ovisensilla on each side on ventral margins and 4 trichoid ovisensilla on dorsal.

***Measurements*** [holotype ♂ (range in 5♂, 5♀), in mm]: BL = 1.87 (1.80–2.07, 1.80–2.13), ThL = 0.93 (0.93–1.00, 0.93–1.00), WL = 2.00 (2.00–2.20, 2.00–2.20), WW = 0.80 (0.80–0.87, 0.73–0.80).

***Indices***: arb = 2/1 (2/1), avd = 0.75 (0.60–1.00), adf = 2.00 (1.50–2.50), flw = 2.00 (1.50–2.50), FW/HW = 0.44 (0.40–0.47), ch/o = 0.10 (0.09–0.10), prorb = 1.00 (0.67–1.00), rcorb = 0.40 (0.33–0.50), vb = 0.30 (0.25 –0.50), dcl =0.70 (0.60–0.80), presctl = 0.60 (0.40–0.64), sctl = 1.18 (1.08–1.25), sterno = 0.60 (0.50–0.78), orbito = 0.80 (0.50–0.67), dcp = 0.27 (0.30–0.36), sctlp = 0.67 (0.67–1.00), C = 2.92 (2.71–3.27), 4c = 1.00 (0.85–1.18), 4v = 2.33 (2.15–2.58), 5x = 1.60 (1.33–1.80), ac = 3.00 (2.20–2.67), M = 0.67 (0.57–0.73), C3F = 0.25 (0.25–0.38).

##### Etymology.

A combination of the Latin words: “*tanaos*” + “*rhinos*”, referring to the developed carina.

##### Distribution.

China (Yunnan).

### Key to examined species of the *riverata* group

**Adults (males)**

**Table:** 

1	Frons reddish brown; clypeus reddish brown	***S. puncticeps* (Okada)**
–	Frons yellowish brown; clypeus yellowish brown	**2**
2	Surstylus bifurcated (Fig. 7A, B)	***S. riverata* (Singh & Gupta)**
–	Surstylus not bifurcated	**3**
3	Dorsal 2 peg-like prensisetae of surstylus separated from each other (Fig. 9A, B)	***S. abdentata* sp. nov.**
–	Surstylus peg-like prensisetae continuous	**4**
4	Gonopods with many finely acanthoid processes	**5**
–	Gonopods lacking finely acanthoid processes	**6**
5	Third to sixth tegites brown (Fig. 5C)	***S. serrateifoliacea* sp. nov.**
–	Third to sixth tegites yellowish (Fig. 5D)	***S. sinuata* sp. nov.**
6	Paramere black	***S. cederholmi* (Okada)**
–	Paramere hyaline	**7**
7	Paramere broadened distally in lateral view (Fig. 10C, D)	***S. platyrhina* sp. nov.**
–	Paramere long in lateral view	**8**
8	Paramere club-shaped	***S. paraclubata* (Sundaran & Gupta)**
–	Paramere finger-like in lateral view	**9**
9	Paramere with fine setae medially (Fig. 13C, D)	***S. tanyrhina* sp. nov.**
–	Paramere lacking fine setae	***S. crocata* (Bock)**

## Supplementary Material

XML Treatment for Scaptodrosophila riverata

XML Treatment for Scaptodrosophila riverata

XML Treatment for Scaptodrosophila puncticeps

XML Treatment for Scaptodrosophila abdentata

XML Treatment for Scaptodrosophila platyrhina

XML Treatment for Scaptodrosophila serrateifoliacea

XML Treatment for Scaptodrosophila sinuata

XML Treatment for Scaptodrosophila tanyrhina
